# Taste the Pain: The Role of TRP Channels in Pain and Taste Perception

**DOI:** 10.3390/ijms21165929

**Published:** 2020-08-18

**Authors:** Edwin N. Aroke, Keesha L. Powell-Roach, Rosario B. Jaime-Lara, Markos Tesfaye, Abhrabrup Roy, Pamela Jackson, Paule V. Joseph

**Affiliations:** 1 School of Nursing, University of Alabama at Birmingham, Birmingham, AL 35294, USA; earoke@uab.edu (E.N.A.); jacksop2@uab.edu (P.J.); 2 College of Nursing, University of Florida, Gainesville, FL 32611, USA; keesharoach@ufl.edu; 3 Sensory Science and Metabolism Unit (SenSMet), National Institute of Nursing Research, National Institutes of Health, Bethesda, MD 20892, USA; rosario.jaime-lara@nih.gov (R.B.J.-L.); markos.tesfaye@nih.gov (M.T.); abe.roy@nih.gov (A.R.)

**Keywords:** pain, transient receptor potential, TRP channel, taste, genomics, capsaicin

## Abstract

Transient receptor potential (TRP) channels are a superfamily of cation transmembrane proteins that are expressed in many tissues and respond to many sensory stimuli. TRP channels play a role in sensory signaling for taste, thermosensation, mechanosensation, and nociception. Activation of TRP channels (e.g., TRPM5) in taste receptors by food/chemicals (e.g., capsaicin) is essential in the acquisition of nutrients, which fuel metabolism, growth, and development. Pain signals from these nociceptors are essential for harm avoidance. Dysfunctional TRP channels have been associated with neuropathic pain, inflammation, and reduced ability to detect taste stimuli. Humans have long recognized the relationship between taste and pain. However, the mechanisms and relationship among these taste–pain sensorial experiences are not fully understood. This article provides a narrative review of literature examining the role of TRP channels on taste and pain perception. Genomic variability in the *TRPV1* gene has been associated with alterations in various pain conditions. Moreover, polymorphisms of the *TRPV1* gene have been associated with alterations in salty taste sensitivity and salt preference. Studies of genetic variations in *TRP* genes or modulation of TRP pathways may increase our understanding of the shared biological mediators of pain and taste, leading to therapeutic interventions to treat many diseases.

Early human hunters and gatherers depended on their ability to distinguish nutritious food from harmful food to avert danger. Gustation and nociception were and continue to be essential for human survival. Gustation, which refers to the sense of taste, gives humans the ability to separate dangerous from nutritious food. Mediated by sensory cells in taste buds located throughout the tongue, taste stimuli can be perceived as sweet, sour, bitter, salty, or savory (umami). [1] In addition, chemosensory nerve endings in the mouth and nose allow for the perception of coolness associated with mint and menthol, and discomfort (burning, irritation, or pain) associated with chili peppers [2]. Capsaicin, an essential chemical in chili peppers, can activate the transient receptor potential (TRP) channels, which transmit the sense of taste and pain [2,3].

Pain-induced activation of the nervous system can alert the body to an actual or potential tissue injury, which may trigger life-saving behaviors such as the flight response, avoidance of painful stimuli, or rest/recuperation behaviors [4]. Perception of pain, or nociception, aids human survival because it allows humans to avoid further injury. Nociception results when nociceptors (nerve endings) in the injured or affected tissue detect and transmit signals to the central nervous system for interpretation. In humans, two primary types of nerves can detect and transmit painful signals: unmyelinated C-fibers and myelinated Aδ- fibers [5]. Recent studies have found that Aδ- and C-fibers depend on electrical signals generated by ion channels, such as TRP channels, to detect and transmit pain [2,5].

TRPs are non-selective ion channels that mediate the fluxes of various types of cations across the cell membrane such as Na^+^, K^+^, Mg^2+^, and Ca^2+^ [2,6]. These channels play essential roles in diverse physiologic processes, but function primarily as gate-keepers for responses to sensory stimuli through the generation of action potentials. Preclinical and clinical studies have identified a wide range of cellular environmental stimuli such as chemicals, temperature, stretch/pressure, osmolarity, and pH that activate TRPs and play a major role in the five primary senses (vision, taste, hearing, smell, and touch) as well as sense of pain [6]. Among these diverse arrays of physiologic functions, the relationship between taste and pain has received limited attention. For example, sucrose is frequently used for the management of acute post-procedural pain in newborn and young infants, yet the mechanism of sucrose-induced analgesia remains unknown [7,8]. More recently, a study of adult volunteers reported that perception of phasic pain may be modulated by both pain and taste. The study showed that sweet taste and smell were associated with lower pain intensity perception and unpleasantness related with phasic pain compared with bitter taste [9].

The purpose of this narrative review is to summarize the role of TRP channels associated with taste and pain and explore potential links between the sense of taste and pain. To achieve the premise of this paper, first, an overview of the structure and functions of TRP channels is presented. Additionally, the role of TRP channels in pain, genetic variability, and analgesia is presented. We will then examine the role of TRPs in taste. Given that hyperalgesia precipitated by an abnormal inflammatory response and oxidative stress, we will discuss the role of TRP in inflammation and oxidative stress. Finally, implications for clinical practice and areas for future research are provided. Many chemicals such as capsaicin and menthol also elicit a sensation of cooling, warmth, pungency, or irritation on the mucous membrane and skin [10]. These sensations, which have been termed chemesthesis and are closely related to taste and pain, will not be discussed in detail in this paper (for review, see Green (2011) [11] and Roper (2014) [10]).

## 1. Overview of TRP Channels

Initially identified in the fruit fly, *Drosophila melanogaster*, TRP channels have been shown to be differentially expressed in the plasma and intracellular membranes of many cells. All TRP channels (in humans and non-humans) belong to a superfamily that shares a fundamental structure consisting of a putative six transmembrane helical protein domains with intracellular C- and N- termini [6,12]. The six-transmembrane structure forms a recurring structural reentrant loop (channel pore) between segment 5 and 6 that principally regulates the movement of cations. Like other ion channels, the regulatory processes of TRP channels require biochemical mechanisms, such as G-protein coupled receptors signaling (e.g., ligand-gated channels and voltage-gated calcium channels), (de)phosphorylation of membrane phospholipids (e.g., phospholipase C (PLC) and phosphatidylinositol 4,5-bisphosphate (PIP2)), and ubiquitination (binding of the ubiquitin to a substrate) [12,13]. Most of these biochemical processes are strongly influenced by changes around the cellular environment. Specifically, most TRP channels play a role in the peripheral nervous system by responding to several intracellular and extracellular stimuli, such as changes in pH, osmolarity/osmolality, temperature, electrolytes, cytokines, injury, and chemicals [6,14]. Once activated, TRP channels function as integrators of these signaling systems to elicit various responses. Some TRP channels are involved in the integration of signals that transmit taste and pain. Table 1 summarizes the primary roles of various TRP subfamilies in pain and taste.

**Figure 1:**
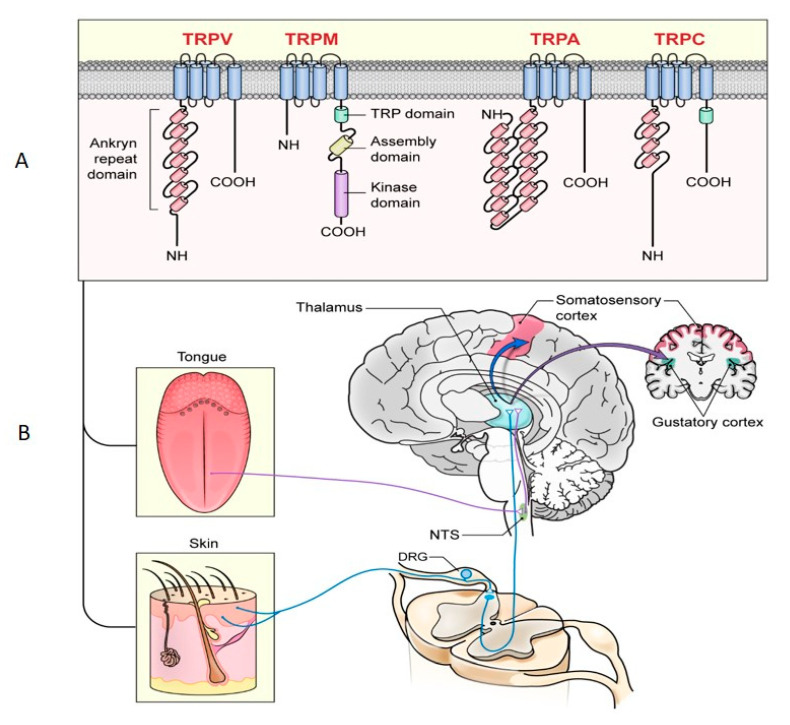
Role of transient receptor potentials (TRPs) in taste and pain sensation: (**A**) TRPs involved in pain and taste perception; (**B**) afferent inputs from nociceptors enter the central nervous system (CNS) via the dorsal root ganglion (DRG) and trigeminal ganglion (TG) for transmission to the cerebral cortex for interpretation. TRPA = transient receptor potential ankyrin; TRPM = transient receptor potential melastatin; TRPV = transient receptor potential vanilloid; TRPC = transient receptor potential-canonical; DRG = dorsal root ganglion; NTS = nucleus tractus solitarius. The blue and purple lines correspond to cranial nerves VII and IX.

About 30 *TRP* genes encode over 100 TRP channels, which are classified into seven subfamilies based on their amino acid sequences and structural conformation [13]. The number of amino acids found at the N terminus and functional motifs varies among the subfamilies. However, subfamily assignment cannot reliably predict the mechanism(s) through which a given TRP is activated and regulated [28]. The seven TRP subfamilies (consisting of one to eight members) that have been identified include canonical (TRPC), vanilloid (TRPV), ankyrin (TRPA), melastatin (TRPM), mucolipin (TRPML), polycystin (TRPP), and non-mechanoreceptor (TRPN). As it relates to this review, members of TRPV, TRPA, TRPC, and TRPM have been determined to be relevant in pain and taste perception (Figure 1A). Thus, our discussion focuses on these subfamilies.

The first mammalian homologs of Drosophila TRP channels identified were the TRP-canonical or classical (TRPC) channels. [29] TRPC channels are Ca^2+^ permeable, non-selective cation channels, which are expressed in many organs such as the heart, kidney, lung, testis, blood vessel, placenta, adrenal gland, and brain [6,13]. TRPC channels regulate many biochemical processes through PIP2 hydrolysis, which results in slow influx of Ca^2+^ and sustained elevation in intracellular Ca^2+^ levels during neuronal transmission [6,29]. Seven (7) distinct members have been identified in the TRPC family: TRPC1, TRPC2, TRPC3, TRPC4, TRPC5, TRPC6, and TRPC7 [6,29]. On the basis of amino acid sequence similarity, the TRPC family is further classified into two subgroups: TRPC1/4/5 and TRPC 3/6/7 [29,30]. TRPC1/4/5 channels are activated by G-protein-couple receptors, which result in downstream activation of PLC, PIP2, and diacylglycerol (DAG), which regulate intracellular Ca^2+^ levels [29,30]. Dysregulation of Ca^2+^ movement across the plasma membrane and TRPC1/4/5 channels dysfunction has been linked with many neurological disorders including pain, anxiety, and depression [29].

The TRPV (vanilloid) subfamily shares about 25 percent amino acid variance with the TRPC subfamily. Initially identified as thermosensitive non-selective cation channels, six (6) distinct TRPV members have been identified in mammals (TRPV1, TRPV2, TRPV3, TRPV4, TRPV5, and TRPV6) with varying cation selectivity [31]. Generally, TRPV1–4 share 40 to 50 percent amino acid sequence variance and are classified as thermo-sensitive, non-selective cation regulators, while TRPV5–6 channels are highly selective to calcium ions [25,31]. TRPV1, the first member of the TRPV family, was identified through expression cloning of the capsaicin receptor and mediates the response to exogenous (e.g., hot chili peppers) and endogenous (e.g., pH < 5.9, inflammatory mediators, and phospholipase C) sensory stimuli, as well as temperature [31,32]. Upon activation by a ligand (e.g., capsaicin), the C-terminal of TRPV channels interacts with calmodulin to regulate the influx through voltage gated Ca^2+^ channels. High levels of intracellular Ca^2+^ have been shown to reduce TRPV5 and TRPV6 ion permeability [33]. TRPV channels are expressed in the membranes of neurons, dorsal root ganglion (DRG), trigeminal ganglion and C-fibers (and to a lesser extent A-δ fibers), epithelial cells of the digestive and respiratory tract, and keratinocytes of the oral mucosa, where they play a role in pain, olfaction, gastrointestinal motility, satiety, and taste [3,32,34,35]. Besides cations, endogenous lipid substances such as PIP2 modulate the activity of TRPV5 and TRPV6 channels [31]. Overall, TRPV channels play an essential role in various conditions, including neuropathic pain, inflammation, diabetes, cancer, and cardiovascular diseases [12].

The transient receptor potential ankyrin 1 (TRPA1) is the only member of the TRPA family expressed in mammals, where it is involved in the sensation of pain; temperature (<17 °C); and chemicals, such as ∆-9-tetrahydrocannabinol (found in marijuana), mustard oil, and bradykinin. [36,37] The presence of 14–18 ankyrin repeat domains in the N-terminal of the TRP protein characterizes this subfamily. The ankyrin repeat domains are ubiquitin rich, short amino acid sequences (up to 33 amino acids) that fold into three-dimensional helix-loop-helix-β-hairpin/loop fold, and create uniquely large intracellular N- and C- termini [2,38]. Mammalian TRPA1 channels are permeable to Ca^2+^ and respond to both electrophilic and non-electrophilic compounds and are expressed in many tissues, including unmyelinated and myelinated C- and Aδ-fibers, lungs, skin, bladder, prostate, pancreas, inner ear, and airway epithelial cells [2]. In most of these tissues, Ca^2+^ (and downstream PLC and PIP_2_) are involved in both the potentiation and desensitization of TRPA1 activity [2,38]. TRPA1 activity has been associated with various acute and chronic pain conditions, cardiovascular disease, obesity, diabetes, respiratory disease, and digestive disorders [38].

The transient receptor potential melastatin (TRPM) shares about 20 percent amino acid identity with the TRPC family and is the largest and most diverse subfamily of the TRP superfamily [39]. TRPM channels are permeable to monovalent (Na^+^ and K^+^) and divalent (Ca^2+^ and Mg^2+^) cations and are expressed in excitable and non-excitable cells in the nervous system, immune system, cardiovascular system, eyes, and keratinocytes [10,12,39]. There are eight (8) members in the TRPM family (TRPM1 to TRPM8), which are classified into four subfamilies (TRPM1/3, TRPM2/8, TRPM4/5, and TRPM6/7) based on amino acid sequence similarities [39]. The primary functions of TRPM channels include immunomodulation, sensory perception, and enzymatic function in response to many stimuli, including temperature, voltage, proteins, lipids, and small endogenous chemicals [12,25,39]. TRPM2 and TRPM3 are activated by endogenous chemicals such as inflammatory mediators, oxidative stress/reactive species, endogenous muscarinic receptor activation, and changes in osmolarity [25]. TRPM8 channel is believed to be voltage regulated and permeable to Ca^2+^, but the mechanism of voltage sensitivity remains unclear [39]. The temperature sensitivity of TRPM8, which is primarily peripheral cold sensitivity, is closely linked to responsiveness to menthol and icilin [14,25,39]. Activation of TRPM8 plays a role in the cooling sensation of menthol in the mouth and mediates the topical analgesic properties of menthol [14]. However, TRPM8 does not appear to affect taste sensation [35]. This may be related to the fact that the cold-sensation mechanism in pain-related inflammation depends on an indirect pathway via preformed G-proteins coupled to TRPM8, rather than the conventional TRPM8 signaling pathway [40].

The C-terminals of some TRPM proteins, such as TRPM2, TRPM6, and TRPM7, have enzymatic activity and are also referred to as “chanzymes”. These chanzymes catalyze hydrolytic cleavage of polyphosphate bonds (hydrolases) and transfer of phosphate groups (kinases) [41]. Together with phospholipase C (PLC) and inositol triphosphate (IP_3_), TRPM5 channels are co-expressed in type II taste receptors and have been shown to play an essential role in the chemical transduction of taste [35,42]. The binding of tastants to taste receptors in type II cells increases the cytosolic Ca2+ concentration, which results in the opening of TRPM5 and the subsequent depolarization of the plasma membrane [35,41,42,43]. Alterations in TRPM channel activity have been associated with inflammatory pain, diabetes, cardiovascular disease, neurodegeneration, and impaired taste transduction [10,22,41].

## 2. The Role of TRP Channels in Pain

For decades, studies have shown that TRP channels have a bi-directional effect on the regulation of crucial pain processes: transduction, transmission, and modulation [2,44,45]. Transduction refers to the processes by which nerve endings detect tissue-damaging injury, while transmission refers to the relay of signal messages from the site of tissue injury to the central nervous system. Neural processes that act to reduce the transmission system and pain perception are referred to as modulation. Thermal, mechanical, or chemical injuries undergo these complex electrochemical processes to reach the cerebral cortex, where the perception of pain is registered [30,46].

Transduction begins with the activation of polymodal specialized nerve endings, known as nociceptors. Given that several ions are expressed in nociceptors, they can be activated by various stimuli. For instance, nociceptors are activated by several chemicals, which are released by tissue injury, including potassium, histamine, and serotonin [5,30]. Moreover, nociceptors are activated by naturally occurring chemicals such as capsaicin in hot chili pepper. Ion channels such as TRP, voltage-gated sodium channels, and acid-sensing ion channels, which are expressed in nociceptors, convert noxious stimuli into electrical signals. For example, TRPV1 can be activated by the capsaicin, acid, and heat. With those different ion channels with different functions, individuals can sense the numerous stimuli. Specifically, TRPs are non-selective cation channels with relatively high Ca^2+^ permeability, which are expressed in peripheral and central nervous system (CNS) terminals, and detect/transduce painful signals [15]. For instance, activation of TRPA1 channels transduces cold stimuli from chemical to electrical signals by regulating sodium and calcium influx [38]. When calcium and sodium ions influx reach the threshold, action potentials are generated and propagated to the central nervous system (CNS) primarily by unmyelinated C- and myelinated Aδ-fibers [47].

Afferent inputs from nociceptors enter the CNS via the dorsal root ganglion (DRG) and trigeminal ganglion (TG) for transmission to the cerebral cortex for interpretation (Figure 1B) [47]. TRPV1 channels are expressed in DRG and TG, where they affect transmission in small and medium nociceptive neurons. Moreover, TRPV1 channels have been identified in nodal and sympathetic ganglia of C- and Aδ- fibers, where they regulate the release of neurotransmitters, including substance P [48,49,50]. Like TRPV1, TRPA1 are expressed centrally in the dorsal horn of the spinal cord where their activation enhances the released glutamate [15]. The release of substance P and glutamate in the spinal cord activates second-order pain transmission neurons, which cross over to the opposite side of the spinal cord before ascending to the brain via spinothalamic and spinoreticular tracts. It is worth noting that TRPM2 and TRPM8 channels are also expressed in DRG and TG, where they regulate noxious nociceptive transmission [51].

Pain modulation refers to the processes by which the body winds up (facilitate) or winds down (inhibit) the transmission of pain signals. Activation of some TRP channels (e.g., TRPV1, TRPA1, and TRPM8) has been reported to play a role in modulating pain perception. During inflammation, bradykinin and other pro-inflammatory substances activate TRPA1 channels in the DRG, which may play a role in inducing hyperalgesia [15,16]. Moreover, TRPA1 amplifies inflammatory pain by enhancing the release of ATP, H^+^, histamine, nerve growth factor (NGF), prostaglandin, and serotonin through protein kinase C [16]. In the spinal cord, TRPM2-mediated infiltration of macrophages and microglia contributes to the pathogenesis of neuropathic pain, while TRPA1-mediated loss of substance P reduces tactile sensitivity in diabetic neuropathy [52,53]. Similarly, excess activation of TRPV1 channels by capsaicin results in TRPV1 de-sensitization, where the resulting antinociception and activation of TRPM8 have been associated with analgesia. Specifically, capsaicin-induced analgesia results from a refractory state potentially involving the depletion of neuropeptides, such as neuropeptide P and counterirritation, where noxious stimuli induce the release of endogenous opioids [54,55]. As the role of TRPs in chronic pain and pain modulation emerges, many TRP channels have been examined as potential therapeutic targets for pain management [14,39]. However, a detailed discussion of the role of TRPs in chronic pain conditions is beyond the scope of this paper.

## 3. *TRP* Genetic Variability and Pain

The *TRPA1* gene is located on chromosome 8q21.11 (GRCh38.p12), and genetic variations in TRPA1 have been associated with various pain syndromes (e.g., familial episodic pain syndrome) [17] and conditions (e.g., neuropathic and inflammatory pain) [51,56]. In familial episodic pain syndrome, a missense mutation in the *TRPA1* gene causes an amino acid substitution (Asp-858-Ser) that alters the functions of this ion channel. As a result, non-noxious stimuli, such as fasting, fatigue, or physical stress, trigger increased pain sensitivity (hyperalgesia) and severe pain in the upper body, which may result in breathing difficulty, abdominal wall stiffness, tachycardia, and sweating [51,57]. The *TRPA1* rs920829 polymorphism (15117G > A) results in a missense variation that is significantly associated with acute pain in sickle cell crises. Compared with patients with the major homozygous GG genotype, those with the minor homozygous AA and heterozygous AG genotypes reported higher rates of acute pain. Moreover, in sickle cell patients, the GCAGG haplotype that contains the rs920829 G allele and rs1025928 C allele is associated with greater utilization of emergency departments and/or acute care centers for pain crisis [58]. In the same sample, study participants with the minor G allele of the *TRPV1* rs222747 polymorphism showed a trend for decreased pain measured by the composite pain index (CPI) [58].

A systematic analysis by Ghosh et al. (2016) examining 2504 genome data suggested high variability in the TRPV family. Mutations in TRPV1 are associated with functional dyspepsia (Ile-315-Met) and decreased risk of asthma (Ile-585-Val). [59] TRPV1 has also been associated with bone cancer pain; TRPV1 inactivation and *TRPV1* gene disruption have been found to decrease pain in animals with bone cancer [60]. Mutations in the *TRPV3* gene result in Olmsted Syndrome, a rare genetic disorder characterized by painful “bilateral mutilating transgredient palmoplantar keratoderma and periorificial keratotic plaques”. [59] TRP4, an osmoreceptor, is activated by innocuous heat (threshold −27 °C), low pH, endocannabinoids, arachidonic acid metabolites, and nitric oxide; TRP4 may also play a role in mechanical hyperalgesia. The *TRPV4* gene is extensively expressed in the brain and spinal cord [52,57]. Mutations in *TRPV4* gene channels result in ion channel dysfunctions and are characterized by motor dysfunctions and neuropathies [57]. These disorders include scapuloperoneal spinal muscular atrophy (Arg-232-Cys) and Charcot–Marie–Tooth disease 2C (Arg-269-His). [57,59] An Ala-563-Thr mutation in TRPV5 results in an increased Ca^2+^ influx and may have an effect on the gating properties of TRPV5 [59]. Okamoto et al. demonstrated that rs8065080 SNP associated with an amino acid substitution, I585V, was significantly related to elevated capsaicin sensitivity [61]. However, the role of these receptors and their relationship to pain and capsaicin is an area of active inquiry.

## 4. TRPs, Pain, Oxidative Stress, and Inflammation

Emerging evidence suggest that TRPs’ ability to detect oxidative stress and other markers of injury or inflammation plays a role in their pain transduction properties [62]. TRPs are able to sense reactive oxygen species [22] and other environmental changes that initiate signal transduction mechanisms indicative of oxidative stress and cell damage (e.g., nerve injury induced by macrophage accumulation and increased oxidative burden) [18,62,63]. Additionally, acute oxidative stress can induce pain by activating pain-sensing neurons, while chronic oxidative stress induces a state of hyperalgesia [64].

TRP channels TRPM2, TRPV1, TRPA1, and TRPC5, are reactive oxygen species (ROS)-sensitive channels that play an integral role in transducing chemical stimuli, including the detection of irritants, pungent chemicals (e.g., capsaicin), and other noxious and nociceptive stimuli [18,63]. TRPA1, TRPV1, and TRPC5 are activated by hydrogen peroxide ROS-induced cysteine modifications [65,66], whereas TRPM2 channels are triggered by more reactive ROS-hydroxyl radicals [67]. Among these TRP channels, TRPA1 has the highest sensitivity to ROS [18].

TRPs’ ability to detect ROS is a protective mechanism that influences cell proliferation and survival. TRPs are associated with apoptosis, where activation of TRP channels by ROS can lead to cell death through sustained calcium influx or through activation of positive feedback mechanisms that further enhance inflammation (e.g., inflammatory cytokines) and tissue damage [19,68,69,70,71,72]. In an in vitro study by Kishimoto et al., capsaicin-induced TRPV1 activation was associated with increased interleukin-8 (IL-8) (an inflammatory cytokine) in human esophageal epithelial cells. This increase in IL-8 was then blocked by the administration of TRPV1 antagonists [70]. More recently, studies have suggested that TRPs may not only play an apoptotic role, but may also play a protective role. For example, two splice variants of TRPM2 have been identified: short unstable TRPM2 (TRPM2-S) and full-length TRPM2 (TRPM2-L). [73] In vitro, TRPM2-L was shown to be protective of oxidative stress by increasing the expression of forkhead box transcription factor 3a (FOXO3a) and superoxide dismutase (SOD) 2, thereby reducing ROS [74,75]. On the other hand, TRPM2-S inhibits the activation of TRPM2-L by oxidative stress [73]. In addition to the potential benefits described above, TRP inhibitors may also have clinically beneficial effects against ROS-related diseases [18,63]. Although TRP agonists/activators can aggravate inflammation and pain under pathophysiological conditions, TRPs can be protective under certain physiological processes. For example, as mentioned previously, TRPM2′s ability to sense ROS triggers transduction pathways that can protect against tissue damage following oxidative stress and ischemia [74], by reducing ROS through increased expression of FOXO3a and SOD1/2 [74,75]. Thus, TRPs are promising targets for pharmacotherapies in the treatment of pain, inflammation, oxidative-stress related diseases, and tissue ischemia.

## 5. The Role of TRP Channels in Taste Sensation

TRP channels are expressed in cells within taste buds, and epithelial cells of the tongue and oral mucosa function to detect stimuli that evoke tastes and chemesthetic sensations [76,77]. Taste gives rise to sensations of sweet, salty, bitter, fat, and savory (umami), while chemesthesis refers to sensations of irritation, pungency, cooling, warmth, and heat produced by chemical stimuli [11]. In many instances, TRP channels function directly as receptors for some chemosensory stimuli, while other TRP channels are downstream effectors of G-protein-coupled sensory receptors [28]. With regards to taste and chemesthesis, TRPV, TRPA, and TRPM have channels that play a role in sensation [10].

TRPM5, expressed in taste cells, plays a significant role in detecting the sweet, umami, and bitter sensations [23]. Temperature is closely linked to taste sensation, and studies have shown that TRPM5 mediates stimuli detection via intracellular cascade that transduces signals to afferent neurons [38,78]. TRPM5 opens through the rises in intracellular calcium, allowing potassium and sodium ions to enter taste cells, and subsequently depolarizing the cell [23]. Animal studies have shown that loss of TRPM5 activation is associated with a reduced to complete loss of sweet, bitter, and umami sensation [23,79]. Moreover, thermal activation of TRPM5 appears to alter taste sensation such that temperature increases in the activity of the TRPM5 channel, which enhances the perception of sweetness [78]. As a result, warm tea may taste sweeter than cold ice cream containing the same amount of sugar [35,78]. More recently, a study identified the role for the sodium-selective TRP channel TRPM4 in taste transduction, showing that TRPM4 and TRPM5 are both involved in taste-evoked signaling. Therefore, a loss of either channel may significantly impair taste, and loss of both channels eliminates the detection of bitter, sweet, or umami stimuli. Hence, both TRPM4 and TRPM5 are needed for transduction of taste stimuli [22]. In addition, although lesser studied, TRPV4 and TRPM8 have also been associated with sensing temperatures and taste by the trigeminal nerves in the tongue [24,77].

The TRPV1 channel is responsive to several chemicals including capsaicin, allicin, gingerol, and alcohol, which are known to modify the activation of the salt stimuli [10,80]. TRPV1t, a TRPV1 channel variant, expressed in the taste buds and epithelial cells of the tongue, plays a role in salty taste transmission by activating an amiloride-insensitive component of the taste receptor [81]. However, the presence of a TRPV1t independent salt taste transduction pathway is also suggested [82]. Animal studies have shown that mice with *TRPV1* gene deletion have an alteration in salt stimuli mediation via the tympani chorda nerve [81] and a higher preference for ethanol consumption [83] than the wild type. More recently, Dias and colleagues examined whether a genetic variation in the *TRPV1* and amiloride-sensitive epithelial sodium channel played a role in salty taste perception in humans [46]. They found that individuals with the T-allele (C > T, Val585Ile) on the *TRPV1* gene SNP (rs8065080) were significantly more sensitive to salty taste than those with the CC genotype [84]. Chamoun and colleagues compared SNPs in taste receptor genes (including *TRPV1*) among 65 adults and 60 children. In children, the C-allele of the *TRPV1* SNP rs4790522 was associated with a higher salt preference compared with A-allele, while in adults, the A-allele of the *TRPV1* SNP rs150908 was correlated with a higher salt sensitivity and lower salt preference [85].

## 6. Implications for Clinical Practice

The role of TRP channels in the transmission of sensory stimuli such as taste and pain remains relatively understudied, yet both taste and pain stimuli are essential for survival and health. Examining the functional mechanism of TRPs at the intersection of taste and pain could aid in understanding the molecular underpinnings of pain and aid in the development of therapeutic interventions.

The roles of TRPs typically associated with taste (e.g., TRPV1 and TRPA1) in mediating sensitivity to pain present a potential opportunity in the development of analgesics. TRPV1 antagonists have been found to reduce pain and inflammation in humans and animal models and are not addictive (unlike opioids and other powerful analgesics with highly addictive properties) [50,55,64,86,87,88,89]. Selective TRPV1 and TRPA1 antagonists reduce pain and inflammation in murine models of pancreatitis [86]. TRP antagonists also reduce thermal and mechanical behavioral responses in animal models of neuropathic pain and inflammation [87,88]. However, in humans, TRP antagonists mainly targeting TRPV1 have been tested for conditions such as dental pain and osteoarthritis, but have shown adverse effects (mainly body temperature dysregulation—hyperthermia), which contributed to the termination of clinical trials [50,90,91]. More recently developed selective TRPV1 antagonists, such as the powerful analgesic NEO6860, did not induce significant temperature dysregulation, making them novel candidates for the treatment of pain [92]. Meanwhile, another TRPV1 agonist, capsaicin, is used in patches [54], topical plaster [55], and other topical formulations to treat site-specific pain (e.g., muscle and joint pain). Capsaicin’s analgesic effects are induced by desensitization and counterirritation, where noxious stimuli enhance the release of endogenous opioids [54,55]. Another TRP that could be targeted for its analgesic effects in humans is TRPA1, which is activated by migraine-inducing agents, suggesting that antagonists may help reduce neuropathic pain and migraine [32,89].

Although the efficacy and nonaddictive properties of TRP-mediated therapies in pre-clinical models make them promising targets for the treatment of pain, drugs targeting TRPs in humans are still under development [50,90,91]. Importantly, current TRP-related pain therapies are not currently used for severe pain (unlike opioids); thus, more potent and effective TRP-related pain therapeutics would have to be developed in order to treat more severe pain. Topical formulations, such as topical capsaicin, are currently being used to provide site-specific analgesia and can be used for mild-moderate pain [54,93,94]. However, more potent systemic drugs are still under investigation. As the opioid epidemic continues to ravish communities in the United States and beyond, it is imperative to develop effective pain therapeutics with nonaddictive profiles, such as TRP-mediated therapies. Further research targeting TRP channels must be conducted to establish their efficacy in the treatment of moderate–severe pain.

## 7. Conclusions

In summary, TRP channels (e.g., TRPA1, TRPM, and TRPV) are located in nerve terminals, dorsal root ganglion, and taste buds, where they play an essential role in pain perception and taste sensation [6,10,15]. Genetic variations of genes coding for TRPs have been associated with changes in taste and pain sensitivity. Additionally, given that activation of TRPs by an inflammatory mediator such as ROS contributes to inflammation and pain, targeting these biological mediators of TRP-related pain could aid in the development of pain treatments. Although the role of TRPs in taste has been a significant area of research, the role of taste-related TRPs for the treatment of pain remains relatively understudied. Thus, studies examining the role of these TRP channels as a potential link between pain and taste are needed. Explicating this relationship may improve our understanding of pain mechanisms and lead to the development of effective and nonaddictive pain therapeutics.

## Figures and Tables

**Table 1:** Role of transient receptor potential (TRP) channels in pain and taste.

Pain		Taste	
TRPA1	▪ Activation transmits nociceptive pain ▪ Inflammatory mediators such as bradykinin indirectly activate TRPA1 channels transmitting inflammatory pain and hyperalgesia [15,16] ▪ Genetic mutation of TRPA1 is associated with familiar episodic pain syndrome [17]	TRPA1	▪ Tastants such as isothiocyanate (mustards), allicin (garlic), and curcumin (turmeric) activate TRPA1 channels [10]
TRPM	▪ Some reactive oxygen species and proinflammatory cytokines activate TRPM2, inducing inflammatory pain and visceral hypersensitivity [18,19] ▪ Neuropeptides activate TRPM3, inducing neurogenic pain [20] ▪ Tissue inflammation and nerve injury can induce cold hypersensitivity (cold-induced pain) and neuropathic pain by activating TRPM8 channels [21]	TRPM	▪ TRPM5 is located on the basolateral surface of the taste receptors and is activated by cytosolic Ca^2+^, increased bitter, sweets, umami, and fatty acids stimuli in taste cells [22,23] ▪ TRPM4 share 40% homology with TRPM5 and may play a role in taste [22] ▪ TRPM8 is activated by several compounds including menthol [24]
TRPV	▪ Inflammatory mediators activate TRPV1 channel inducing inflammatory, neuropathic, and visceral pain [25] ▪ Activation of TRPV2 channels evoked inflammatory pain behaviors ▪ Activation of TRPV3 channels induces inflammatory pain signals [26] ▪ TRPV4 channels are sensitized by inflammatory mediators, and also transduce visceral and neuropathic pain [6]	TRPV	▪ Species such as capsaicin (chili pepper), acids (vinegar), allicin (garlic), gingerol, and piperine (black pepper) activate TRPV1 in taste buds [27]

TRPA1 = transient receptor potential ankyrin 1; TRPM = transient receptor potential melastatin; TRPV = transient receptor potential vanilloid.
